# A multicenter analysis of implantable monitoring device-based diagnosis of supraventricular arrhythmia post patent foramen ovale closure: the OCCL-ILR study

**DOI:** 10.3389/fcvm.2025.1541923

**Published:** 2025-04-04

**Authors:** Stijn Lochy, Alvise Del Monte, Xavier Galloo, Andreea Motoc, Daniele Plein, Kurt Hermans, Frauke Gorré, Julie Colas-Florial, Liesbeth Rosseel, Philip Muyldermans, Maarten Pauwelyn, Pieter-Jan Palmers, François Delvoye, Evelyne Wirix, Jonas Podevyn, Bram Roosens, Philippe Unger, Steven Droogmans, Jean-François Argacha, Sylvie De Raedt, Gian Battista Chierchia, Bernard Cosyns

**Affiliations:** ^1^ Department of Cardiology, Vrije Universiteit Brussel (VUB), Universitair Ziekenhuis Brussel (UZ Brussel), Brussels, Belgium; ^2^ Heart Rhythm Management Center, Postgraduate Program in Cardiac Electrophysiology and Pacing, Vrije Universiteit Brussel (VUB), Universitair Ziekenhuis Brussel (UZ Brussel), Brussels, Belgium; ^3^ Department of Cardiology, Algemeen Ziekenhuis Sint-Lucas, Gent, Belgium; ^4^ Hartcentrum Aalst, AZorg, Aalst, Belgium; ^5^ Department of Cardiology, Algemeen Ziekenhuis Delta, Roeselare, Belgium; ^6^ Department of Cardiology, Clinique CHC MontLégia, Luik, Belgium; ^7^ Department of Neurology, Clinique CHC MontLégia, Luik, Belgium; ^8^ Department of Neurology, Universitair Ziekenhuis Brussel (UZ Brussel), NEUR Research Group, Vrije Universiteit Brussel (VUB), Brussels, Belgium

**Keywords:** PFO (patent foramen ovale), atrial fibrillation, structural cardiology, occlutech PFO device, implantable loop recorder (ILR)

## Abstract

**Background:**

Recent data suggest that the true incidence of atrial fibrillation (AF) after patent foramen ovale (PFO) closure has probably been underestimated, and may differ according to the type of closing device used.

**Objectives:**

On the basis of continuous rhythm monitoring with an implantable device, this study aims to assess the incidence of  supraventricular arrhythmia following PFO closure with the Occlutech PFO device.

**Methods:**

This is a multicentric analysis of consecutive PFO closure patients treated with an Occlutech device between 01/01/2019 and 20/03/2024, with an implantable loop recorder (IRL) (or a pacemaker or implantable cardioverter defibrillator) implanted for at least 3 months preceding the procedure, and with available follow-up for at least 1month post procedure. Primary endpoint was the incidence of patients with new onset supraventricular arrhythmia (AF, atrial flutter or any supraventricular tachycardia) lasting >30 s, post PFO closure.

**Results:**

A total of 59 patients met the inclusion criteria. Patients were monitored (95% with ILR) during 284 days (IQR 241.5–374) before, and for 422 days (IQR 237–776) post PFO closure. Supraventricular arrhythmia post PFO closure was reported in 18 patients (31%), with median time-interval until arrhythmia occurrence of 16.5 days (IQR 13–21). A total of 88 supraventricular arrhythmia events (96.6% AF) were documented during follow-up. In 94.4% of patients with supraventricular arrhythmia, new-onset arrhythmia occurred in the first 45 days after PFO closure. Six patients (33.3%) with supraventricular arrhythmia post PFO closure, presented AF episodes beyond 60 days after PFO closure.

**Conclusions:**

In this multicenter retrospective analysis of patients undergoing percutaneous PFO closure with the Occlutech PFO device, implantable continuous rhythm monitoring devices were able to diagnose new-onset supraventricular arrhythmia (97% AF) after PFO closure in 31% of patients. While 94% of new-onset supraventricular arrhythmia events occurred in the first 45 days post-procedure, one-third of patients with arrhythmia post PFO closure presented AF episodes beyond 60 days post procedure.

## Introduction

While being a highly prevalent condition in the general population, there is currently solid evidence linking patent foramen ovale (PFO) to cryptogenic stroke. Several randomized controlled trials (RCT's) (1–3) showed a significant reduction in recurrent cryptogenic stroke by PFO closure compared to medical therapy. Consequently, PFO closure has become an established therapy for stroke prevention.

However, patient selection for PFO closure poses several challenges due to the complex interplay of factors that influence the procedure's benefits and risks. PFOs are found in ∼25% of the general population, but not all are causally linked to stroke. Identifying whether the PFO is related to the paradoxical embolism, or if the stroke resulted from another cause (such as occult atrial fibrillation) can be difficult. The use of implantable loop recorders (ILR's) significantly increases the detection rate of AF, and their systematic use has even be advocated in the work-up of stroke patients being considered for PFO closure (4, 5).

However, AF may also complicate PFO closure, with PFO-related AF most commonly occurring in the first 1–2 months after the procedure, with some authors taking 45 days as the cut-off for PFO-device related AF (6). The incidence of AF after PFO closure was initially reported to be in the range of 2.5%–5%, but these data were derived from AF diagnosis based on clinical symptoms (6). Recent trial data (7–9) from PFO patients monitored with ILR and/or external loop recorders post-procedure, have indicated a higher incidence, with AF occurring in approximately 20% of patients, often with a subclinical course.

Moreover, the incidence of AF after PFO closure seems to differ according to the type of closing device used (10). Herein, we report the results of a retrospective, multicenter registry of patients treated with the Occlutech PFO device, undergoing systematic rhythm monitoring using implantable continuous monitoring devices both before and after the procedure.

## Methods and materials

### Study design and population

All consecutive patients, treated with percutaneous PFO closure in 4 non-academic hospitals and 1 academic hospital between 01/01/2019 and 20/03/2024, were retrospectively reviewed for inclusion. Diagnosis of PFO was established with transthoracic and/or transesophageal echocardiography, by confirmation of significant right-to-left shunting using agitated saline injection, with and without Valsalva maneuver. A severe shunt was characterised as passage of more than 20 microbubbles. Indication for PFO closure was established by the local heart team involving a neurologist, with secondary prevention of cardio-embolic ischemic stroke, peripheral embolic events and platypnea-orthodeoxia syndrome as potential indications for closure. Patients were eligible if they were carrying an IRL (or a pacemaker or implantable cardioverter defibrillator) for at least 3 months before the PFO closure procedure. ILR use was generally part of an AF rule-out strategy. The PFO closure procedure was performed according to local standard procedures; both general or local anesthesia (+sedation) could be used, as well as imaging guiding with (mini) transesophageal or intracardiac echocardiography. Implantation of an Occlutech PFO device was mandatory for inclusion. Device size was decided by the implanting operator, with or without balloon sizing.

After the procedure, patients needed to have heart rhythm follow-up with ILR (or pacemaker/ICD) for at least 1 month, but results of ILR (or pacemaker/ICD) monitoring beyond 1 month were also collected. All ILR (or pacemaker/ICD) EGM tracings from both before and after PFO closure were reviewed by three independent cardiologists (including an electrophysiologist) for arrhythmia analysis (core laboratory analysis). Atrial fibrillation was defined as significant if an episode of irregularly irregular rhythm without distinct *P* waves lasting >30 s was documented on the EGM. For all patients, relevant demographic, clinical and procedure-related data were also collected from the patient file. This study was carried out in accordance with the Declaration of Helsinki. The ethical committee of the University Hospital of Brussels approved the study, which was subsequently also approved by the local ethics committees of the other enrolling institutions. Patient's consent was waived because of the retrospective character of the trial.

### Study endpoints

The primary endpoint of the study was the proportion of patients with new onset, significant (lasting >30 s) supraventricular arrhythmia [AF, atrial flutter or any supraventricular tachycardia (SVT)] post PFO closure.

The secondary endpoints were the proportion of patients with new onset, significant (lasting >30 s) supraventricular arrhythmia (AF, atrial flutter, or any SVT), in the first 30 days, between 30 and 60 days, and beyond 60 days post PFO closure.

### Device

The Occlutech PFO device is a self-centering device made of nitinol wires forming two flexible retention discs. Its efficacy and safety have been shown in several trials (11, 12), and it is currently one of the most frequently used devices for PFO closure.

Four device sizes are available, with the right atrial disc ranging from 18 to 35 mm. Compared to the Abbott Amplatzer PFO Occluder, it has a ball-forceps connection for attaching the device to the pusher, and it has a larger left atrial disc for each size.

### Statistics

Statistical analyses was performed using R software (R Foundation for Statistical Computing, Vienna, Austria) and IBM SPSS Statistic for Windows (Armonk, NY: IBM Corp.). Normal distribution of continuous variables was assessed by Shapiro-Wilk test. Continuous variables were presented as medians [interquartiles (IQR)] for non-normally distributed variables, or means [+- standard deviation (SD)] for normally distributed variables. Categorical variables were presented as percentages.

Continuous variables were compared using the unpaired *t* test, Mann–Whitney or the Wilcoxon signed-rank test as appropriate. Categorical variables were compared using the chi-square test or Fisher's exact test, as appropriate. A *P* value less than 0.05 was considered significant. Kaplan–Meier survival curves were constructed to evaluate the time to the onset of new supraventricular arrhythmia.

## Results

A total of 63 patients met the inclusion criteria, but after a secondary read of all EGM's by the core lab, 4 patients were excluded because of the presence of atrial fibrillation before PFO closure, leaving a final study population of 59 patients.

The baseline population characteristics are described in Table 1. There were no significant differences in the clinical profile of patients who developed, vs. those who did not develop supraventricular arrhythmia. The median age of the population was 55 years (IQR 45–59), with a slight male predominance (54%). The median CHA2DS2-Vasc and ROPE score were calculated based on available baseline characteristics, and were 3 (IQR 2–4) and 6 (IQR 5–7), respectively. Thirty-three patients (56%) were treated with the 27–30 mm Occlutech PFO device, 19 patients (32%) with the 23–25 mm device and 7 patients (12%) with the 31–35 mm device.

**Table 1:** Baseline population characteristics.

Characteristic	Total (*N* = 59)	No supraventricular arrhythmia post PFO closure *N* = 41 (69%)	Supraventricular arrhythmia post PFO closure *N* = 18 (31%)	*P* value
Age (year)	55 (45–59)	54 (48–58)	58 (50–63)	0.3
Female sex	27 (46%)	21 (51%)	6 (33%)	0.2
Body mass index (kg/m²)	26 (24–29)	26 (24–29)	24 (22–28)	0.2
PFO closure indication				
TIA/ischemic stroke	57 (97%)	39 (95%)	18 (100%)	>0.9
Peripheral embolism	2 (3%)	2 (5%)	0 (0%)	>0.9
History of recurrent TIA/ischemic stroke	22 (37%)	13 (32%)	9 (50%)	0.2
eGFR >60 ml/min/1.73 m^2^	58 (98%)	40 (98%)	18 (100%)	>0.9
Smoking	12 (20%)	8 (20%)	4 (22%)	>0.9
Arterial hypertension	25 (42%)	16 (39%)	9 (50%)	0.4
Diabetes mellitus	4 (7%)	2 (5%)	2 (11%)	0.6
Heart failure	3 (5.1%)	2 (4.9%)	1 (5.6%)	>0.9
Coronary artery disease	4 (6.8%)	4 (9.8%)	0 (0%)	0.3
Peripheral artery disease	1 (1.7%)	1 (2.4%)	0 (0%)	>0.9
CHA_2_DS_2_VASc score	3 (2–4)	3 (2–4)	3 (2–4)	0.8
Venous thrombo-embolism	4 (6.9%)	3 (7.5%)	1 (5.6%)	>0.9
Thrombophilia	6 (11%)	3 (7.7%)	3 (17%)	0.4
ROPE score	6 (5–7)	6 (5–7)	6 (4–6)	0.3
Severe shunt at baseline	36 (61%)	26 (63%)	10 (56%)	0.6
Indexed left atrial volume (ml/m²)	24.6 ± 6.8	24.4 ± 6.2	25.1 ± 8.5	0.383
Size occlutech PFO occluder				0.7
23–25 mm	19 (32%	12 (29%)	7 (39%)	
27–30 mm	33 (56%)	23 (56%)	10 (56%)	
31–35 mm	7 (12%)	6 (15%)	1 (5%)	

Values are median (IQR) or *n* (%). Indexed left atrial volume is mean ± standard deviation.

PFO, patent foramen ovale; ILR, implanted loop recorder; RoPE, risk of paradoxical embolism; GFR, glomerural filtration rate; TIA, transient ischaemic attack.

Continuous rhythm monitoring was obtained mainly using ILR (95%), with the Medtronic Reveal LINQ™ being the most frequently used device (68% of ILR use). Monitoring with a pacemaker or ICD was available in 5% of the patients (Table 2). Patients were monitored during a median period of 284 days (IQR 241.5–374), before PFO closure was performed.

**Table 2:** Monitoring device characteristics.

Monitoring device	
ILR	56 (95%)
Pacemaker	2 (3%)
ICD	1 (2%)
Type ILR device	
Abbott CONFIRM Rx™ ICM	4 (7%)
Biotronik biomonitor III	14 (25%)
Medtronic reveal LINQ™	38 (68%)

Values are *n* (%).

ILR, implanted loop recorder; ICD, implantable cardioverter defibrillator.

**Figure 1:**
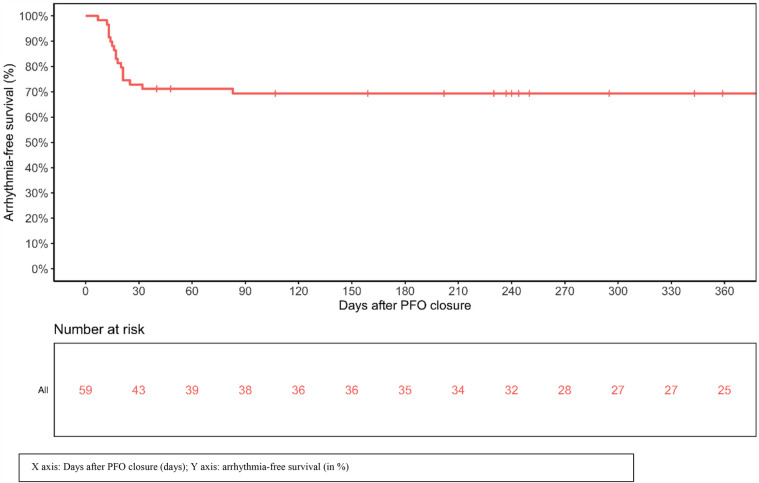
Arrhythmia-free survival post PFO closure.

After PFO closure, rhythm monitoring was available for 422 days (IQR 237–776). Analysis of implanted device monitoring, showed supraventricular arrhythmia after PFO closure (AF, atrial flutter or SVT) in 18 patients (31%) (Table 3). A total of 88 supraventricular arrhythmia events were documented during follow-up, with a median duration of 4.5 min (IQR 2–106.5): 96.6% (*N* = 85), 2.3% (*N* = 2), and 1.1% (*N* = 1) were identified as AF, atrial flutter, and SVT, respectively. The 3 non-AF supraventricular arrhythmia episodes all occurred in the first 30 days after closure. All supraventricular arrhythmia events beyond 30 days were AF. Patients experienced a median number of 2 (IQR 1–5.5) arrhythmia episodes, with a median cumulative supraventricular arrhythmia burden per patient of 144 min (IQR 13.75–357). The median time-interval between PFO closure and supraventricular arrhythmia occurrence was 16.5 days (IQR 13–21). Figure 1 shows the arrhythmia-free survival post PFO closure. Supraventricular arrhythmia occurred in the first 30 days after PFO closure in 88.9% (16/18) of patients, between 30 and 60 days after PFO closure in 5.6% (1/18), and beyond 60 days after PFO closure in 5.6% (1/18). Notably, 94.4% of new supraventricular arrhythmia episodes occurred in the first 45 days post procedure.

**Table 3:** Monitoring results.

Duration of device monitoring pre PFO closure (days)	284 (241.5–374)
Duration of device monitoring post PFO closure (days)	422 (237–776)
Supraventricular arrhythmia post PFO closure	18/59 (31%)
Total number of supraventricular arrhythmia events in study population	88
Type of supraventricular arrhythmia	
AF	85/88 (96.6%)
Aflutter	2/88 (2.3%)
SVT	1/88 (1.1%)
Median duration of supraventricular arrhythmia event in study population (minutes)	4.5 (2–106.5)
Number of episodes of supraventricular arrhythmia per patient	2 (1–5.5)
Total cumulative burden of supraventricular arrhythmia events per patient (minutes)	144 (13.75–357)
Time-interval between PFO closure and first supraventricular arrhythmia (days)	16.5 (13–21)
Evaluation in the first 30 days post PFO closure	
First episode of supraventricular arrhythmia	16/18 (88.90%)
Number of patients experiencing supraventricular arrhythmia in the first 30 days post PFO closure/all patients experiencing supraventricular arrhythmia post PFO closure	16/18 (88.90%)
Number of supraventricular arrhythmia episodes per patient (with supraventricular arrhythmia)	3 (1–3)
Total number of supraventricular arrhythmia episodes	59
Median duration of supraventricular arrhythmia episode (minutes)	4 (2–107)
Evaluation between 30 and 60 days post PFO closure	
First episode of supraventricular arrhythmia	1/18 (5.55%)
Number of patients experiencing supraventricular arrhythmia between 30 and 60 days post PFO closure/all patients experiencing supraventricular arrhythmia post PFO closure	7/18 (38.89%)
First supraventricular arrhythmia in the first 45 days post PFO closure	17/18 (94.4%)
Number of supraventricular arrhythmia episodes per patient (with supraventricular arrhythmia)	2 (1–2.5)
Total number of supraventricular arrhythmia episodes	15
Median duration of supraventricular arrhythmia episode (minutes)	4 (2–59)
Evaluation beyond 60 days of PFO closure	
First episode of supraventricular arrhythmia	1/18 (5.55%)
Number of patients experiencing supraventricular arrhythmia beyond 60 days post PFO closure/all patients experiencing supraventricular arrhythmia post PFO closure	6/18 (33.33%)
Number of supraventricular arrhythmia episodes per patient (with supraventricular arrhythmia)	1.5 (1–2.75)
Total number of supraventricular arrhythmia episodes	14
Median duration of supraventricular arrhythmia episode (minutes)	63 (5–344.75)

Values are median (IQR) or *n* (%).

PFO, patent foramen ovale.

**Figure 2:**
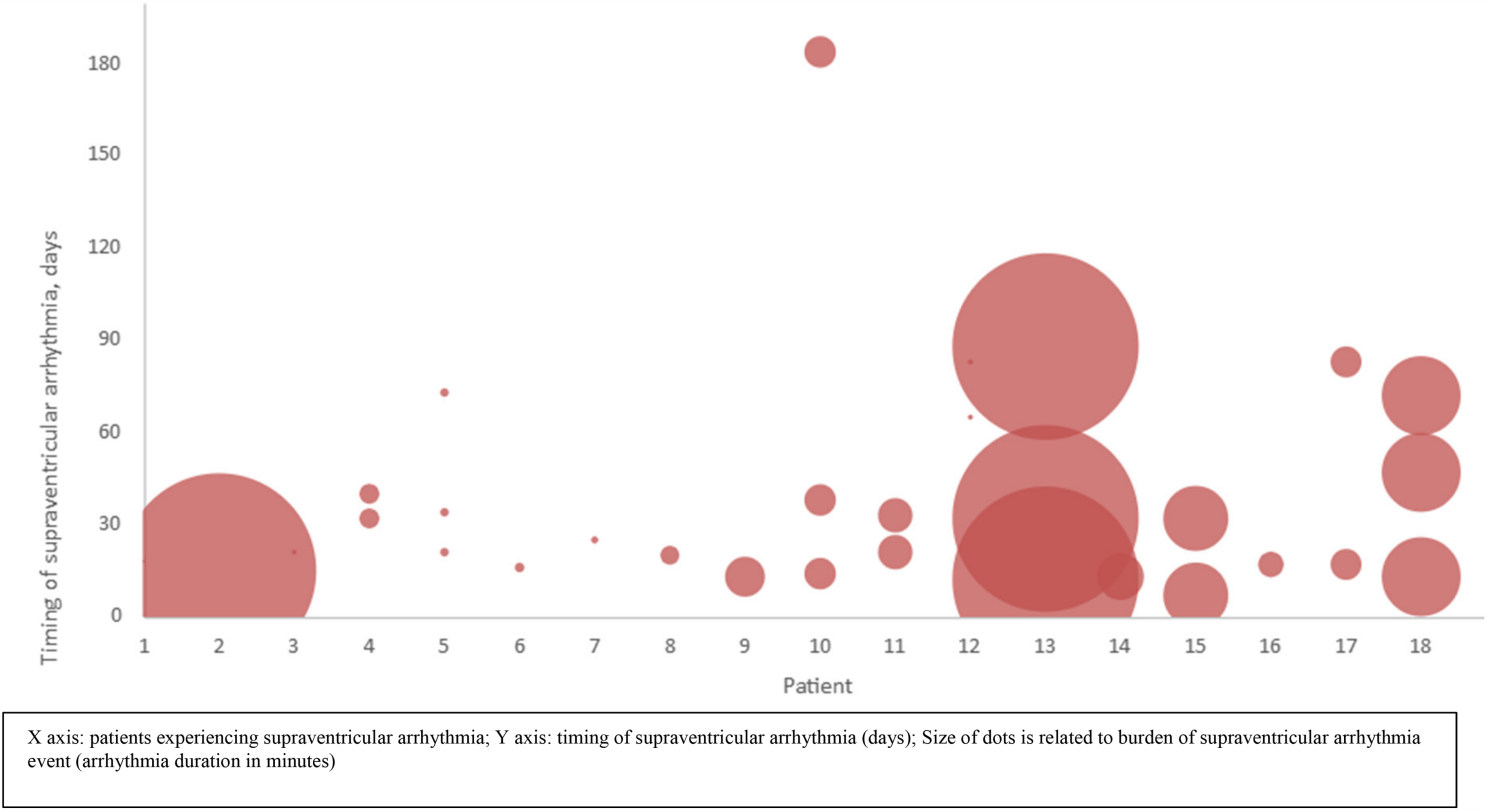
Supraventricular arrhythmia burden and timing per patient.

A total of 59 arrhythmia episodes were documented in the first 30 days after PFO closure, 15 episodes occurred between 30 and 60 days, and 14 beyond 60 days. The number of supraventricular arrhythmia episodes per patient declined from 3 (IQR 1–3) in the first 30 days, to 2 (IQR 1–2.5) between 30 and 60 days and 1.5 (IQR 1–2.75) beyond 60 days. While new-onset AF beyond 60 days post-procedure was diagnosed in only 1 (5.55%) of patients, AF recurrence beyond 60 days was detected in 5 (27.8%) patients with previously detected post-procedural supraventricular arrhythmia. Therefore, a total of 6 (33.3%) patients with supraventricular arrhythmia post PFO closure, presented AF episodes beyond 60 days after PFO closure. The median supraventricular arrhythmia episode duration was both 4 min in the first 30 days (IQR 2–107) and between 30 and 60 days (IQR 2–59) post closure; beyond 60 days median arrhythmia duration was 63 min (IQR 5–344.75). Figure 2 depicts the supraventricular arrhythmia burden and timing per patient.

Immediately post-PFO closure, 98.3% of patients were treated with dual anti-platelet therapy (aspirin + clopidogrel) (Supplementary Table 1). After diagnosis of supraventricular arrhythmia post PFO closure, only 55.6% of these patients were switched from antiplatelet therapy to a direct anticoagulant (DOAC).

After PFO closure, baseline anti-arrhythmic treatment with betablockers or amiodarone was prescribed in respectively 13.57% and 1.69% of patients (Supplementary Table 1). In 6 of 18 patients with supraventricular arrhythmia post PFO closure (33.3%), a new anti-arrhythmic drug was initiated during follow-up, with sotalol being the most frequently prescribed agent (57.14%).

In only 1 patient, PFO closure was associated with a peri-procedural complication (pseudo-aneurysm of the A. femoralis). For more details on hemorrhagic, thromboembolic or cardiovascular complications during follow-up, we refer to Supplementary Table 2.

## Discussion

Increasing data suggest that the true incidence of AF after percutaneous PFO closure is underestimated. Earlier studies reported an incidence of AF of 2.5%–5%, but these were mainly based on the occurrence of symptomatic AF, or AF was an incidental finding during follow-up. The primary finding of this first multicenter study, utilizing continuous rhythm monitoring with implantable devices both before and after PFO closure, is the detection of supraventricular arrhythmia in 31% of patients following closure with a single device type, the Occlutech PFO device. AF was by far the most frequent arrhythmia reported, accounting for nearly 97% of arrhythmia events.

These results are in line with other recent reports, using long-term continuous rhythm monitoring. Guedeney et al. (7) reported an incidence of 21% of new onset supraventricular arrhythmia post PFO closure using continuous monitoring devices, but only 37% were monitored with an ILR. In the ILR subpopulation, an incidence of 29% was also observed. Only 8.4% of these patients had an ILR implanted prior to the procedure as part of an initial AF rule-out strategy. More recently, a study including 35 patients monitored with ILR reported an incidence of new onset AF post PFO closure of 31% (9). Notably, the median duration of rhythm monitoring prior to PFO closure was 184 days (IQR: 98; 280 days).

With a median of 284 days (IQR 241.5–374 days) of monitoring before PFO closure, and with a per-protocol core-lab second read of all EGM's pre- and post-PFO closure, our study allowed AF to be more thoroughly excluded, allowing to characterize a very selected population of true “PFO-related stroke patients”.

Our observed ILR-based incidence rate of AF is in line with previous studies using ILR for AF detection in patients with cryptogenic stroke. The CRYSTAL AF and the STROKE AF trial (4, 13), which both used ILR, observed a similar 6-fold increase rate of AF diagnosis following cryptogenic stroke, in comparison to clinical follow-up.

Whereas a CHA2DS2-Vasc score of at least 2 (because of history of stroke) theoretically warrants anticoagulation, the recent NOAH-AFNET 6 and ARTESIA trials (14, 15) suggested that anticoagulation may be less beneficial in patients with subclinical, device detected AF. Interestingly, in our population only 55.6% of patients with supraventricular arrhythmia post PFO closure were switched to a DOAC. It remains speculative if the treating physician considered these arrhythmia episodes to be of low thrombo-embolic risk, or if this was related to arrhythmia underdiagnosis. Hence, more clinical outcome data are needed in this specific subpopulation of patients with AF post PFO closure, which would help to optimize anti-thrombotic strategy. The long-term follow-up results of the PFO-AF trial (16) might shed more light on this issue.

In this study, 94% of new-onset supraventricular arrhythmia occurred in the first 45 days post procedure, with a median time-interval of 16.5 days. This timing is strongly suggestive of a device-related pathogenesis. PFO device-related AF is generally considered benign and self-limiting, resulting from local irritation, metallic component interference, tissue stretch, and perhaps nickel hypersensitivity (6).

Data from randomized trials have shown that 72% of these new-onset AF episodes spontaneously resolve within 45 days after PFO closure (17). Our data are in line with this observation. However, while only 1 patient (5.55%) experienced new-onset AF beyond 60 days post-procedure, 5 other patients with supraventricular arrhythmia arising in the first 45 days were detected with recurrent AF beyond 60 days. Thus, among the patients experiencing supraventricular arrhythmia post procedure, up to 33% suffered from AF beyond 60 days post procedure. In addition, the burden of AF (number of AF episodes, and median duration of AF episodes) remains considerable beyond 60 days (Figure 2).

While anti-arrhythmic treatment at baseline (or initiation during follow-up) was not infrequent in our study population, its effect on occurrence of supraventricular arrhythmia post PFO closure remains unclear. Interestingly, in the recently published AFLOAT trial, flecainide did not prevent atrial arrhythmia after PFO closure (18).

Nevertheless, the observations on early and late occurrence of AF post PFO closure could potentially be relevant for the management of these patients; prolonged (beyond 45–60 days) monitoring, and possibly anticoagulation, might be considered when a diagnosis of supraventricular arrhythmia is made. Future research is warranted focusing on identifying risk factors for delayed AF occurrence (and recurrence) post PFO closure. In this regard, despite lacking statistical significance in this population with relatively low sample size, early supraventricular arrhythmia burden and number of episodes could be particularly interesting for further exploration.

Despite being widely used in clinical practice, there is only sparse prior data on Occlutech PFO device-related arrhythmia. The device was also not used in the landmark randomized PFO trials. In their long-term observational follow-up data of the Occlutech PFO device, Snijder et al. reported a 6.6% incidence of new onset of clinical (thus without ILR) supraventricular arrhythmia post PFO closure, among which 2.6% occurred more than 2 years after closure (19). The Occlutech PFO device has also been compared with the Amplatzer PFO device, which shares several design similarities. This comparison included also a Holter monitoring at 1-month follow-up (20). In this observational registry, supraventricular arrhythmias occurred more frequently with the Amplatzer PFO device (17% vs. 9%). In the most recent trial from Guedeney et al. (7) using continuous rhythm monitoring, Occlutech PFO devices were used alongside mostly Abbott and Gore PFO devices, but the use of different device types (including also multi-fenestrated and ASD devices) hampered interpretation. Future trials should aim to compare the incidence rate of AF of different PFO devices.

### Study limitations

This study may have a selection bias increasing the inclusion of patients with predisposition to AF, inherent to the retrospective design with inclusion limited to patients with ILR implantation before PFO closure.

The sample size was relatively small, despite the multicentric design of the study, which reduced the statistical power to perform regression analyses. As a result, we were unable to adjust for potential confounders or to explore independent predictors of early or late supraventricular arrhythmia onset, which may affect the generalizability and robustness of our findings.

## Conclusion

In this multicenter retrospective analysis of patients undergoing percutaneous PFO closure with the Occlutech PFO device, implantable continuous rhythm monitoring devices were able to diagnose new-onset supraventricular arrhythmia (of which 97% AF) after PFO closure in 31% of patients. While 94% of these new-onset arrhythmia events occurred in the first 45 days post-procedure (median 16.5 days), one-third of patients with supraventricular arrhythmia post PFO closure presented AF episodes beyond 60 days after the procedure. Future research should focus on the prevention and management of late-occurring AF post PFO closure.

## Data Availability

The raw data supporting the conclusions of this article will be made available by the authors, without undue reservation.
