# Supplementary material for: A multicenter analysis of implantable monitoring device-based diagnosis of supraventricular arrhythmia post patent foramen ovale closure: the OCCL-ILR study
Source: Front Cardiovasc Med. 2025 Apr 4;12:1541923. doi: 10.3389/fcvm.2025.1541923 (PMC12006060; doi:10.3389/fcvm.2025.1541923)
Supplement: Supplementary file 1 [file Table1.pdf]

**Supplementary table1. Pharmacological treatment post PFO closure**

| | |
|---|---|
| Baseline anti-thrombotic treatment immediately post PFO closure<br>- Aspirin + clopidogrel<br>- Clopidogrel + OAC | <br>58/59 (98.3%)<br>1/59 (1.7%) |
| Switch to OAC or DOAC after diagnosis of supraventricular arrhythmia | 10/18 (55.6%) |
| Anti-thrombotic treatment at last clinical follow-up<br>- Aspirin<br>- Clopidogrel<br>- Aspirin + clopidogrel<br>- DOAC<br>- OAC<br>- None | <br>27/59 (45.76%)<br>20/59 (33.91%)<br>5/59 (8.47%)<br>5/59 (8.47%)<br>0/59 (0%)<br>2/59 (3.39%) |
| Baseline anti-arrhythmic treatment immediately post PFO closure<br>- Betablockers<br>- Amiodarone<br>- None | <br>8/59 (13.56%)<br>1/59 (1.69%)<br>50/59 (84.75%) |
| Start (or switch) of anti-arrhythmic treatment during follow-up<br>- Patients with supraventricular arrhythmia post PFO closure<br>- Patients without supraventricular arrhythmia post PFO closure | <br>6/18 (33.3%)<br>1/41 (2.44%) |
| New anti-arrhythmic drug introduced during follow-up<br>- Betablockers<br>- Sotalol<br>- Amiodarone | <br>2/7 (28.57%)<br>4/7 (57.14%)<br>1/7 (14.29%) |
| Values are n (%).<br>PFO: patent foramen ovale<br>DOAC: direct anticoagulants<br>OAC: oral anticoagulation | |
