# Supplementary material for: A multicenter analysis of implantable monitoring device-based diagnosis of supraventricular arrhythmia post patent foramen ovale closure: the OCCL-ILR study
Source: Front Cardiovasc Med. 2025 Apr 4;12:1541923. doi: 10.3389/fcvm.2025.1541923 (PMC12006060; doi:10.3389/fcvm.2025.1541923)
Supplement: Supplementary file 2 [file Table2.pdf]

Supplementary table2. Complications: peri-procedural and during follow-up

Peri-procedural/in-hospital complications post PFO closure	
- Vascular access site (arterial pseudo-aneurysm)	1/59 (1.7%)
- None	58/59 (98.3%)
Hemorrhagic complications during follow-up	
- Traumatic subarachnoid bleeding	1/59 (1.7%)
- Gynaecological bleeding	1/59 (1.7%)
- None	57/59 (96.6%)
Thrombo-embolic complications during follow-up	
- Pulmonary embolism	1/59 (1.7%)
- Ischemic stroke	1/59 (1.7%)
- None	57/59 (96.6%)
Cardiovascular complications during follow-up	
- Second degree Av block	2/59 (3.4%)
- None	57/59 (96.6%)
Values are n (%).<br>PFO: patent foramen ovale<br>AV block: atrioventricular block	
